# The effects of myeloablative or non-myeloablative total body irradiations on intestinal tract in mice

**DOI:** 10.1042/BSR20202993

**Published:** 2021-03-02

**Authors:** Shengyun Zhu, Jing Liang, Feng Zhu, Xue Zhang, Mengdi Xu, Kai Zhao, Lingyu Zeng, Kailin Xu

**Affiliations:** 1 Institute of Blood Diseases, Xuzhou Medical University, Xuzhou, Jiangsu 221002, China; 2 Department of Hematology, Affiliated Hospital of Xuzhou Medical University, Xuzhou, Jiangsu 221002, China; 3 The Key Laboratory of Bone Marrow Stem Cell, Jiangsu 221002, China

**Keywords:** intestinal tract, myeloablative irradiation, p38 MAPK, radiotherapy, short chain fatty acid, ZO-1

## Abstract

Acute radiation injury caused by high-dose radiation exposure severely impedes the application of radiotherapy in cancer management. To deeply understand the side effects of radiation on intestinal tract, an irradiation murine model was applied and evaluated. C57BL/6 mice were given 4 Gy non-myeloablative irradiation, 8 Gy myeloablative irradiation and non-irradiation (control), respectively. Results demonstrated that the 8 Gy myeloablative irradiations significantly damaged the gut barrier along with decreasing MECA32 and ZO-1. However, a slight increase in MECA32 and ZO-1 was detected in the 4 Gy non-myeloablative irradiations treatment from day 5 to day 10. Further, the irradiations affected the expression of P38 and JNK mitogen-activated protein kinase (MAPK) but not ERK1/2 MAPK signal pathway. Moreover, irradiation had adverse effects on hematopoietic system, altered the numbers and percentages of intestinal inflammatory cells. The *IL-17/AhR* had big increase in the gut of 4 Gy irradiation mice at day 10 compared with other groups. Both 8 Gy myeloablative and 4 Gy non-myeloablative irradiation disturbed the levels of short-chain fatty acids (SCFAs) in intestine. Meanwhile, high dosage of irradiation decreased the intestinal bacterial diversity and altered the community composition. Importantly, the fatty acids generating bacteria *Bacteroidaceae* and *Ruminococcaceae* played key roles in community distribution and SCFAs metabolism after irradiation. Collectively, the irradiation induced gut barrier damage with dosages dependent that led to the decreased p38 MAPK and increased JNK MAPK, unbalanced the mononuclear cells (MNCs) of gut, disturbed intestinal bacterial community and SCFAs level.

## Introduction

Malignant tumors have become the second leading cause of deaths worldwide and pose a serious threat to public health. There are at least 50% malignant tumor patients treated by radiotherapy, 25% of which are able to survive [1]. Therefore, radiotherapy becomes an indispensable treatment for the majority of malignant tumors [1,2]. However, acute radiation injury caused by high-dose radiation exposure severely limits the widespread application of radiotherapy because of the indiscriminate harm to normal tissues and organs [3,4]. The hematopoietic system and intestinal tract are highly sensitive to ionizing radiation and easily injured, especially in treating for hematologic tumor, abdominal tumor and gynecological tumor [5,6].

Hematopoietic stem cell transplantation is recognized as a fundamental solution for hematological malignant tumor [7,8]. Total body irradiation (TBI) is used in humans mostly for the treatment of acute lymphatic leukemia as conditioning of transplant. Briefly, myeloablative irradiations or non-myeloablative irradiations coupled with multidrug chemotherapy are required to clear the recipient’s tumor cells and empty bone marrow cavity for implantation. However, exposure to overdose of irradiation will limit the proliferation of crypt epithelial cells, impact the regeneration of villi epithelial cells, and further destroy the integrity of the epithelium and the intestinal microbiota, eventually leading to the fatal complications including endogenous sepsis or multiorgan failure [9,10]. After TBI of 12 Gy or more in murine (C3Hf/Sed//Kam), death from hematopoietic depletion occurred within 10 days [11]. Rescue with syngeneic bone marrow cells after TBI also increased the median lethal dose for gut lethality by 3 Gy [12]. However, death after doses greater than 19 Gy is primarily due to intestinal stem cell depletion and lethality is not modified by hematopoietic reconstitution with bone marrow transplantation [11,12].

Extensive studies focus on the prevention and treatment of radio-induced intestinal injury, but no effective therapeutic measures appear so far [13]. Till now, the only FDA-approved radio-protective drug (WR2721, Amifostine, marketed as Ethyol® by Med Immune, Gaithersburg, MD) is used clinically [14], but still not free of toxicity. Hence, it is urgent to utilize a stable and reliable animal experimental model to deeply understand radio-induced intestinal injury. In the present study, we applied 8 and 4 Gy as myeloablative and non-myeloablative TBI in C57BL/6 mice to evaluate the effects of these irradiations on intestinal tract.

## Materials and methods

### Radiation injury mouse model

C57BL/6 mice and BALB/c mice, male, aged 6–8 weeks, were purchased from Beijing Vital River Laboratory Animal Technology Co., Ltd., China. The mice were housed under specific pathogen-free conditions with ambient temperature at 20 ± 2°C, a 12-h/12-h light/dark cycle and all animal experiments took place at the Experimental Animal Center of Xuzhou Medical University. For samples collection, animals were first anesthetized by intraperitoneal injection 10% chloral hydrate solution and then killed by gently taking off the neck. All animal care and experimental procedures followed ethical standards of animal use and were approved by Xuzhou Medical University.

C57BL/6 mice were exposed to different dosages of ^60^Co irradiation (4 Gy non-myeloablative irradiation, 8 Gy myeloablative irradiation and non-irradiation as control), and killed at days 5 and 10. CTR (*n*=5), represents samples from normal C57BL/6 mice; 4D5 (*n*=5) and 4D10 (*n*=5), represent samples from C57BL6 mice at day 5 or 10 after 4 Gy ^60^Co irradiation, respectively and 8D5 (*n*=5), represents samples from C57BL6 mice at day 5 after 8 Gy ^60^Co irradiation.

BALB/c mice were exposed to ^60^Co irradiation, grouped into 7.5 Gy myeloablative irradiation mice (TBI group) and normal mice without irradiation (Normal group). Mice were killed at day 5 and cecal contents were collected for the gut bacterial diversity assay.

### Frozen tissue immunofluorescence analysis

The harvested intestinal tissues were divided into segments and immediately embedded in O.C.T. Compound (Tissue Freezing Medium, Servicebio, Wuhan, China) on dry ice. Tissue was cut into 8-μm sections using freezing microtome (Leica CM1950, Leica, Germany), then fixed in 4% paraformaldehyde for 15 min at room temperature, permeabilized with 0.3% Triton-X-100 in Triton Buffer Solution for 10 min, blocked with 5% gout serum in TBS-0.05% Tween-20 for 1 h and stained with primary antibodies in blocking buffer at 4°C overnight. Primary antibodies included: anti-ZO-1 (Proteintech Group, Inc., 21773-1-AP, 1:1000 dilution), anti-MECA32 (Novus Biologicals, CO, NB100-77668, 1:1000 dilution). The next day, the sections were stained with appropriate secondary antibodies in blocking buffer for 45 min and nuclei were counterstained with DAPI.

### Real-time quantitative PCR

The intestinal tissues were lysed with Automatic sample rapid grinding machine (JXFSTPRP-24, Jingxin, Shanghai) and mRNA was extracted by TRIzol (Thermo Fisher Scientific, Shanghai, China) as described in previous methods [15]. Total RNA was reverse transcribed using the Transcriptor First Strand cDNA synthesis kit (Roche Life Science, Basel, Switzerland) and analyzed on Roche Light Cycler 480 with SYBR Green I master (Roche Life Science, Basel, Switzerland). All data were normalized to the expression of *β-actin* and the fold difference in expression relative to that of *β-actin* is shown. The primer sequences were as follows: *β-actin* (F-ATGGAGGGGAATACAGCCC; R-TTCTTTGCAGCTCCTTCGTT), *ZO-1*(F-GCAGACTTCTGGAGGTTTCG; R-CTTGCCAACTTTTCTCTGGC), *MECA-32* (F-CGTCAAGGCCAAGTCGCT; R-TGGATCAATGGGTGGAGGGT), *IL-17* (F-ATCCACCTCACACGAGGCACAA; R-AGATGAAGCTCTCCCTGGACTCAT), *AhR* (F-AGCCGGTGCAGAAAACAGTA; R-CCAGGCGGTCTAACTCTGTG).

### Protein extraction and Western blotting from intestinal tissue

Intestinal tissue homogenates were prepared in cold RIPA lysis buffer containing protease inhibitor cocktail and PMSF by Automatic sample rapid grinding machine. Equal amounts of proteins were separated using 12% SDS/PAGE. Following transfer on to NC membrane (General Electric Company, Buckinghamshire, United Kingdom) for 1.5 h, the membranes were blocked and incubated with the primary antibodies P38 (1:2000 dilution; cst 8690t), phos-P38 (1:1000 dilution; cst 4511t), ERK1/2 (1:1000 dilution; cst 4659), phos-ERK1/2 (1:1000 dilution; cst 4370), phos-SAPK/JNK (1:1000 dilution; cst 4668) from Cell Signaling Technology (Danvers, MA), antibody α-tublin (1:4000 dilution; 11224-1-ap) from Proteintech Group (Wuhan, China) and JNK123 antibody (1:1000 dilution; AF6318) from Affinity Biosciences (Cincinnati, OH) overnight on shaker at 4°C. After washing, membranes were incubated with suitable HRP-conjugated secondary antibody for 1 h at room temperature and bands were visualized using ECL chemiluminescence reagents. The intensities of each protein band were measured using ImageJ software.

### Peripheral blood, lymph nodes cell counts, and isolation of intestinal intraepithelial lymphocytes

Blood was directly obtained from the orbital sinus and collected in pre-coated K_2_EDTA-containing tubes. The mesenteric lymph nodes (LNs) were harvested, grinded and filtered through a 200-mesh sieve. Cell counting assay was performed with those collected cells, including white blood cells (WBCs), red blood cells (RBCs) and platelets (PLTs).

For intestinal intraepithelial lymphocytes (IELs), the intestines were flushed with PBS^-Ca/-Mg^ after dissection of fat and mesenteric tissue and excision of Peyer’s patches, and cut into 1-cm pieces. The intestines pieces were incubated with 20 ml HBSS (5 mM EDTA, 3% FBS, 1% P/S, and 1 mM DTT) for 15 min on a shaker at 37°C, single cells were filtered and collected. Residual intestinal pieces were added with 20 ml fresh HBSS and repeated collection. Collected single cells were then purified on a 40 and 80% Percoll solution (General Electric Company, Buckinghamshire, United Kingdom).

### Flow cytometry analysis

For analysis of the percentages of CD45^+^CD4^+^ T cell, CD45^+^CD8^+^ T cell, CD45^+^CD4^+^CD25^+^ T cell, CD45^+^CD8^+^CD25^+^ T cell, CD45^+^F4/80^+^ cell, CD45^+^CD11b^+^ cell and CD45^+^MHCII^+^ cell, the IELs from small intestine or colon and mononuclear cells (MNCs) from LN cells obtained above were stained for tube 1: CD45 (APC Cy7, clone 30-F11), CD4 (FITC, clone RM4-5), CD8A (PE, clone 53-6.7), CD25 (APC, clone PC61) or tube 2: CD45 (APC Cy7, clone 30-F11), F4/80 (PE, clone BM8), MHCII (PerCP-CY5.5, clone M5/114.15.2), CD11b (APC, clone M1/70) antibody at room temperature for 20 min and sorted on a BD FACSAria device. All antibodies were from BD Biosciences (Franklin Lakes, NJ) except anti-F4/80 and anti-CD11b from Biolegend (San Diego, CA).

### Short-chain fatty acids detection

Mice cecal contents were homogenized in distilled water and filtrated via 0.22-μm membrane for short-chain fatty acids (SCFAs; acetic acid, butyric acid, valeric acid) measurement. A high-performance liquid chromatography (HPLC, Agilent1260 Infinity Quaternary, CA) equipped with a SPD-20A UV/Vis detector monitored at 215 nm for SCFAs. The SCFAs were simultaneously analyzed through a HPX-87H column (300 × 7.8 mm) (Bio-Rad, Hercules, CA) and kept at 55°C, flow 0.5 ml.min^−1^, eluent 0.045 N H_2_SO_4_ with 6% acetonitrile (v/v) [16].

### DNA extraction, 16S rRNA gene amplicon sequencing and bioinformatics analysis

The total DNA was extracted from approx. 0.2 g feces from killed BALB/c mice (with or without myeloablative irradiation) by the QIAamp DNA Stool Mini Kit (cat# 51504, QIAGEN, Hilden, Germany). The gut-microbial 16S rRNA gene was amplified using primers 515F (5′-GTGCCAGCMGCCGCGGTAA-3′) and 926R (5′-CCGTCAATTCMTTTGAGTTT-3′). The purified PCR products were loaded for sequencing on the MiSeq Illumina platform (Tiny Gene Bio-Tech Co., Ltd., Shanghai, China). The raw fastq files were processed and analyzed following general protocol as primary publication [35].

### Statistical analysis

Datasets are presented as mean ± SEM. At least three independent experiments were performed for each assay. For comparison between vehicle and different dosages of TBI, data were assessed by one-way ANOVA using GraphPad Prism software. *P*-values are less than 0.05 represent statistical significance. The statistical calculations of microbial dataset were based on R (version 3.4.2) software packages.

## Results

### Myeloablative irradiations induce the destruction of gut epithelial barrier

**Figure 1:**
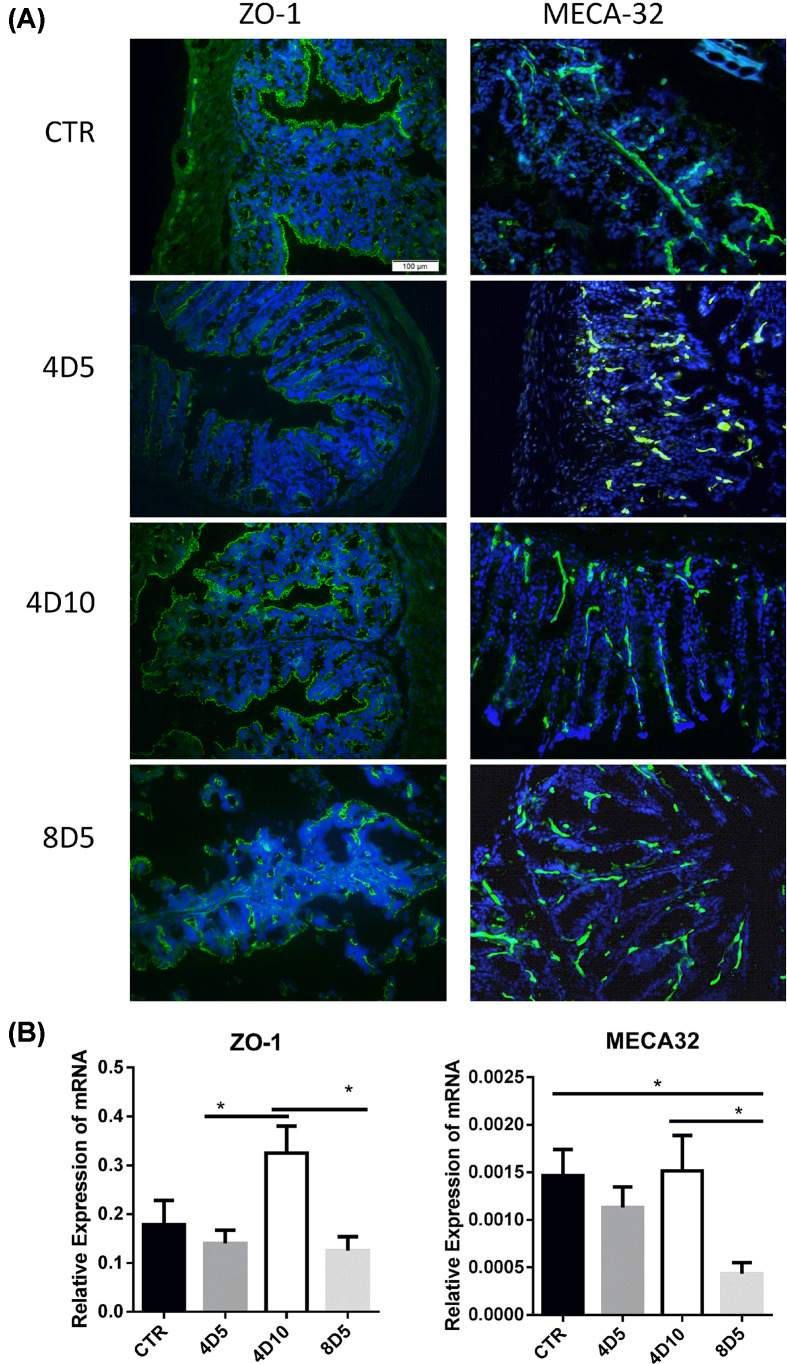
Myeloablative irradiations induce the destruction of gut epithelial barrier (**A**) Representative confocal images of ZO-1 (left) and MECA32 (right) staining in the intestinal tissue from untreated or irradiated mice, Blue: DAPI. Original magnification ×200. Scale bar = 100 μm. All images shared the same scale bar in one combined image. (**B**) The mRNA expression levels of *ZO-1* and *MECA32* in intestine tissues of mice in the four groups was examined by RT-qPCR. *P<0.05. CTR, represents samples from normal C57BL/6 mice as control; 4D5 and 4D10, represent samples from C57BL/6 mice at day 5 or 10 after 4 Gy ^60^Co irradiation respectively; 8D5, represents samples from C57BL/6 mice at day 5 after 8 Gy ^60^Co irradiation. Abbreviation: RT-qPCR, real-time quantitative PCR.

To establish the TBI mice model, C57BL/6 mice were treated with 4 Gy non-myeloablative TBI, 8 Gy myeloablative TBI and unirradiated treatment (CTR), respectively. Figure 1 illustrates the impact of irradiations on the integrity of gut epithelial barrier and gut vascular endothelium. In our study, the mice treated by 4 Gy TBI had 100% survival but only 20% survival was detected in the 8 Gy TBI mice at day 10. The 8 Gy group exhibited a striking reduction and discontinuous distribution of ZO-1 (tight junction protein) at day 5 compared with other groups (Figure 1A). The 4 Gy group exhibited a slightly decrease in ZO-1 at day 5, but almost recover to normal level at day 10. This phenomenon may be a sign that the gut epithelial barrier was being repaired after the low dosage of irradiation. On the other hand, both 4 and 8 Gy irradiation reduced MECA32 (pan-vascular endothelial cell antigen) at day 5, however, mice with 4 Gy irradiation displayed improved MECA32 expression at day 10. Furthermore, the mRNA levels of *ZO-1* and *MECA32* (Figure 1B) showed a similar trend as the protein levels (Figure 1A). These results indicated that low dosage of irradiation induced mild gut injury but can be rescued in a short time (10 days in the study), this response was confirmed by the increasing ZO-1 in epithelial cells and MECA32 in vascular endothelial cells.

### Irradiations affected the mitogen-activated protein kinase signal pathway

**Figure 2:**
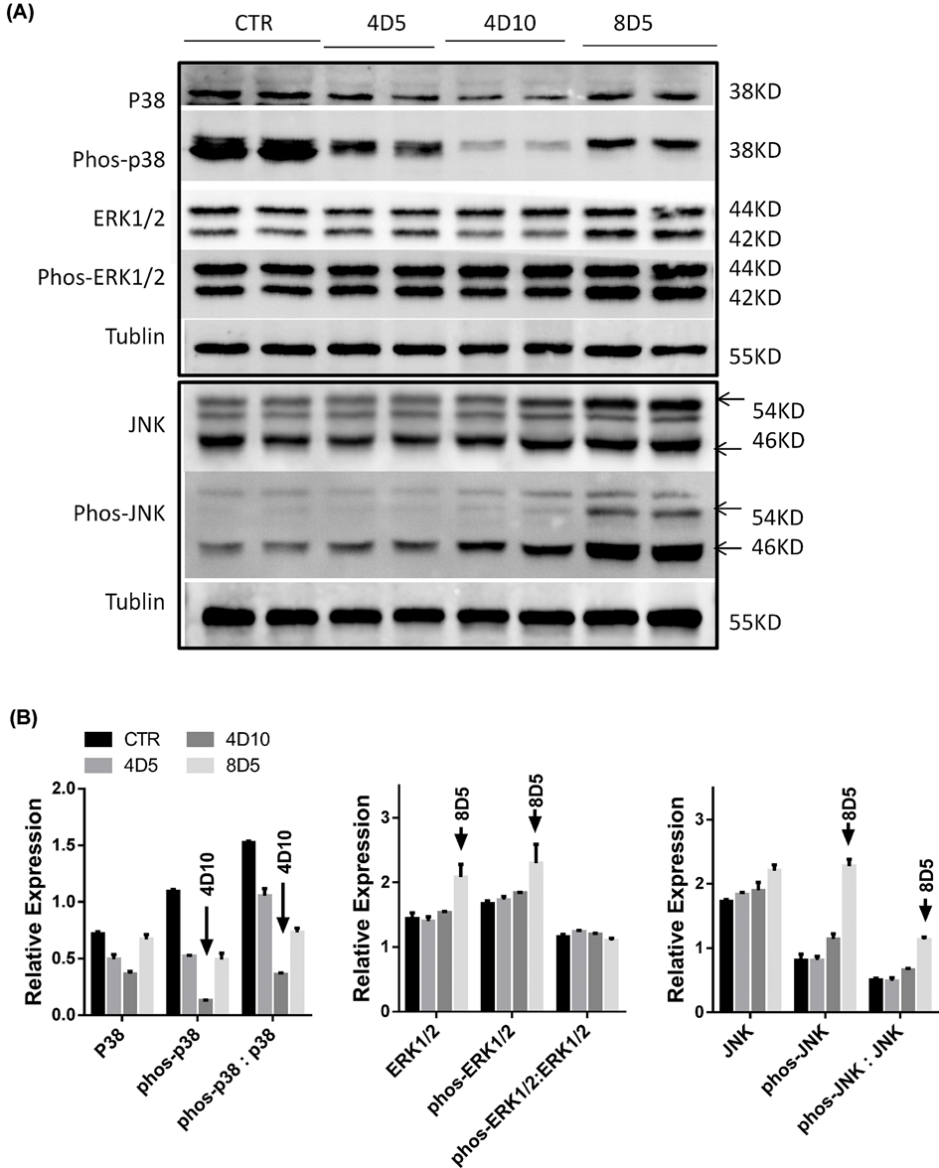
Irradiations affected the expression of intestinal MAPK signal pathway-related molecules (**A**) The different expressions of intestine tissue p38, phos-p38, ERK1/2, phos-ERK1/2, JNK and phos-JNK proteins in untreated or irradiated mice were analyzed by Western blotting. (**B**) Bar graph showed the gray levels of MAPK-related molecules as mentioned in (A).

Mitogen-activated protein kinase (MAPK) signal pathway is closely linked with the damage and repair of intestinal tissues [17,18]. Figure 2 demonstrates how the irradiations affected the protein expressions of MAPK signal pathway in intestinal tract. Compared with normal group, the levels of phos-p38 were significantly down-regulated facing 4 and 8 Gy TBI treatments, meanwhile, 4 Gy treatment reduced further the phos-p38 level from days 5 to 10. In addition, the stress-activated protein kinase/Jun-amino-terminal kinase (SAPK/JNK), is potentially activated by a variety of environmental stresses including γ radiation. As we found, 8 Gy TBI significantly increased the level of phos-JNK protein and the ratio of phos-JNK to JNK protein, there was no big change in 4 Gy group related to normal group. On another hand, the 4 and 8 Gy irradiated mice did not obviously attenuate the ratio of phos-ERK1/2 to ERK1/2 (Figure 2B), although both ERK1/2 and phos-ERK1/2 in the 8D5 group showed a slight increase. This phenomenon may suggest a stress response after fatal radioactive intestinal injury. Therefore, the irradiation may mainly decrease the level of phos-p38 protein and the ratio of phos-p38 to p38 protein, as well as increase the level of phos-JNK protein and the ratio of phos-JNK to JNK protein in gut tissue.

### Irradiations affect hematopoietic system and intestinal inflammation *in vivo*

**Figure 3:**
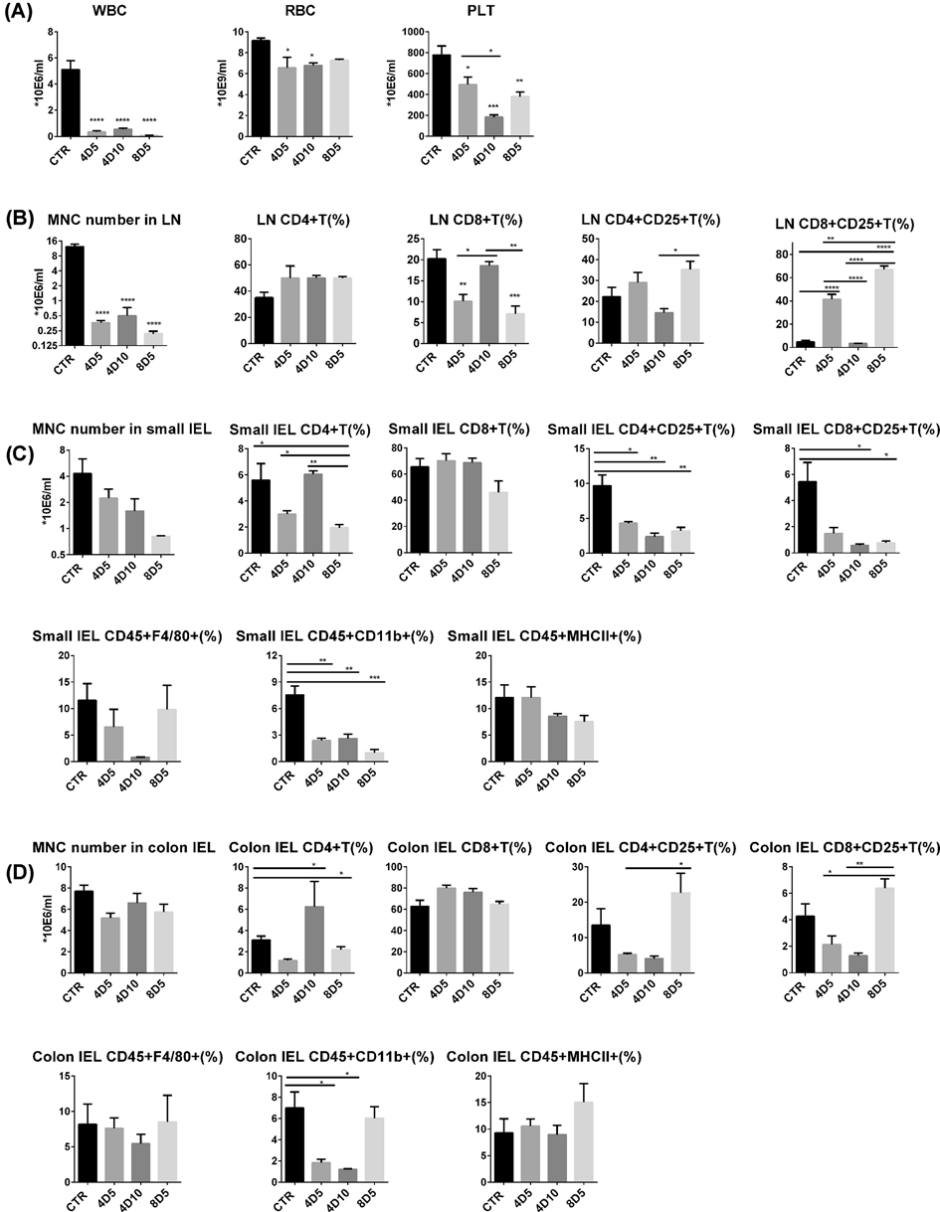
Irradiations can inhibit hematopoietic function and influence the development of intestinal inflammation (**A**) The numbers of WBC counts, RBC counts and PLT counts in peripheral blood were quantified on days 5 and 10 after TBI. (**B**) The number of MNCs in mesenteric LNs was quantified by cell counter and the percentages of CD4^+^ T cells, CD8^+^ T cells in CD45^+^ lymphocytes and the percentage of CD4^+^CD25^+^ T cells and CD8^+^CD25^+^ T cells were analyzed by flow cytometry. The numbers of IELs and the percentages of CD4^+^ T cells, CD8^+^ T cells, CD4^+^CD25^+^ T cells, CD8^+^CD25^+^ T cells, CD45^+^F4/80^+^ cells, CD45^+^CD11b^+^ cells and CD45^+^MHCII^+^ cells in small intestine (**C**) and in colon (**D**). *P<0.05, **P<0.01, ***P<0.001, ****P<0.0001.

It has been confirmed that irradiation-induced myelosuppression is manifested as the decrease in bone marrow activity followed by a decline of peripheral blood cells [5,19]. To examine the adverse effects of TBI on the hematopoietic system, we investigated the abundance of different types of hematopoietic cells in peripheral blood, mesenteric LNs and intestinal tract in the irradiated mice. The mice receiving 4 and 8 Gy TBI exhibited a big decrease in WBCs (*P*<0.0001), RBCs (*P*<0.05) and PLTs (*P*<0.05) in peripheral blood, and PLTs continuously decreased from days 5 to 10 compared with the control group (Figure 3A). Moreover, the numbers of MNCs in the mesenteric LNs of irradiated mice exhibited significantly decreased (*P*<0.0001) (Figure 3B), while number of IELs in small intestine and colon showed a slight decrease than the control group (Figure 3C,D).

Both active T cells and partial active B cells contain abundant CD25 molecules, which is participation of high affinity IL-2R to active T cells. In order to explain the inflammatory stimulation effect of myeloablative and non-myeloablative irradiations on the intestinal tract, we investigated the proportion of CD4^+^ T cells, CD8^+^ T cells, CD4^+^CD25^+^ T cells and CD8^+^CD25^+^ T cells in the mesenteric LNs and IELs of intestinal tract. For the mesenteric LNs (Figure 3B), the percentage of CD8^+^ T cells decreased obviously after 4 Gy (*P*<0.01) and 8 Gy (*P*<0.001) irradiation at day 5 compared with control group. There was a big increase in CD8^+^CD25^+^ T cells in mesenteric LNs after TBI at day 5, however, it dropped rapidly at day 10.

For the small intestinal tissue (Figure 3C), the irradiations significantly decreased the percentage of CD4^+^ T cells (at day 5). Nevertheless, there was no significant difference in the percentage of intestinal CD8^+^ T cells in all groups. Irradiations significantly reduced the percentages of intestinal CD4^+^CD25^+^ T cells and CD8^+^CD25^+^ T cells at days 5 and 10 (*P*<0.01), which were different from that in mesenteric LNs. Meanwhile, irradiations also decreased the percentage of CD45^+^CD11b^+^ cells compared with control group, reduced CD45^+^F4/80^+^ cells in 4D10 group. For the colon (Figure 3D), the percentage of CD4^+^ T cells and CD8^+^ T cells showed similar trend with that in small intestine after irradiation. Interestingly, compared with 4 Gy treatment, the 8 Gy irradiation significantly increased the percentages of CD4^+^CD25^+^ T cells and CD8^+^CD25^+^ T cells at day 5 (*P*<0.05). Similar rising trend was observed in 8D5 group in mesenteric LNs. Moreover, the percentage of CD45^+^CD11b^+^ cells decreased after 4 Gy irradiations but slightly increased after 8 Gy irradiation. The above results indicated that the irradiation reduced the total numbers of blood cells and MNCs in intestinal tract, and changed the percentages of various immune cells. This may explain why the irradiation-treated mice could not show spontaneous recovery within 10 days.

### Irradiation impacts on IL-17, AhR and SCFAs

**Figure 4:**
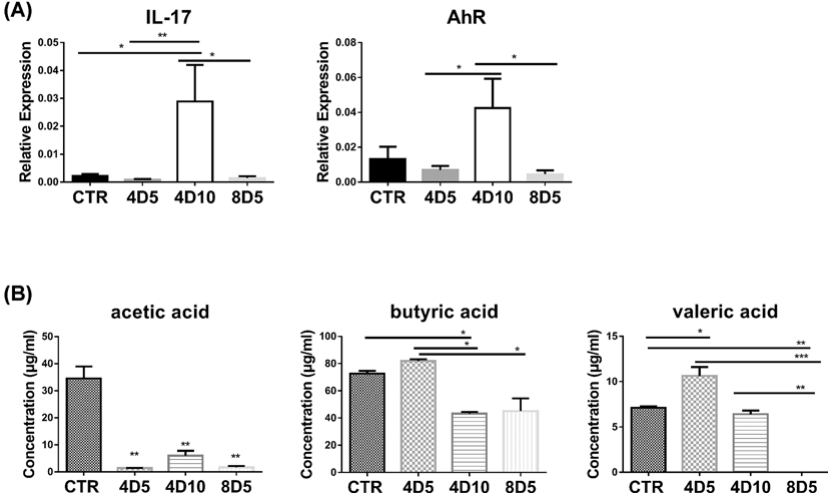
Irradiations had effects on intestinal IL-17/AhR and commensal microbiota-derived SCFAs (**A**) The expression levels of intestinal *IL-17* and *AhR* in mice with or without irradiation were examined by RT-qPCR. (**B**) The concentration of SCFAs (acetic acid, butyric acid and valeric acid) in different fecal samples was analyzed by HPLC. *P<0.05, **P<0.01, ***P<0.001. Abbreviation: RT-qPCR, real-time quantitative PCR.

IL-17/AhR signaling is essential for regulating mucosal host defense against invading pathogens and intestine repairing [15,16,20]. The AhR expressed highly in intestinal mucosa, which can regulate the proliferation and function of helper T lymphocytes 17 (Th17) and regulatory T lymphocytes (Tregs) in peripheral blood [21,22]. Compared with control group, the mRNA expression of intestinal *IL-17* and *AhR* did not show big difference after 4 and 8 Gy TBI at day 5, but the two factors increased dramatically after 4 Gy TBI at day 10 (Figure 4A). The results indicated that 4 Gy non-myeloablative irradiation-induced gut injury might start to repair at approx. day 10 after TBI via IL-17/AhR signaling pathway.

A growing number of evidence suggested that the gut microbiota can release SCFAs [23], and these metabolites play a key role in intestinal micro-ecology including regulate host health and immune response [24,25]. Given the intestinal tract is highly sensitive to irradiation, we further determined the effects of 4Gy-TBI and 8Gy-TBI on gut commensal-derived SCFAs. Results showed that both non-myeloablative and myeloablative irradiations greatly reduced the levels of acetic acid compared with the control group (Figure 4B). Meanwhile, lower level of butyric acid was found in the 8D5 and 4D10 groups, which means the metabolism of butyric acid is not sensitive to irradiation. In addition, the 4 Gy non-myeloablative irradiations had higher level of valeric acid at day 5, but the 8 Gy myeloablative irradiations obviously decreased valeric acid at day 5. These results suggested that gut metabolites were sensitive to irradiation, and we can also speculate that some key gut microbiota was still active under the low dosage of irradiation or non-myeloablative irradiations to sustain the intestinal micro-ecology.

### Irradiations’ effects on microbial diversity and distribution in gut

**Figure 5:**
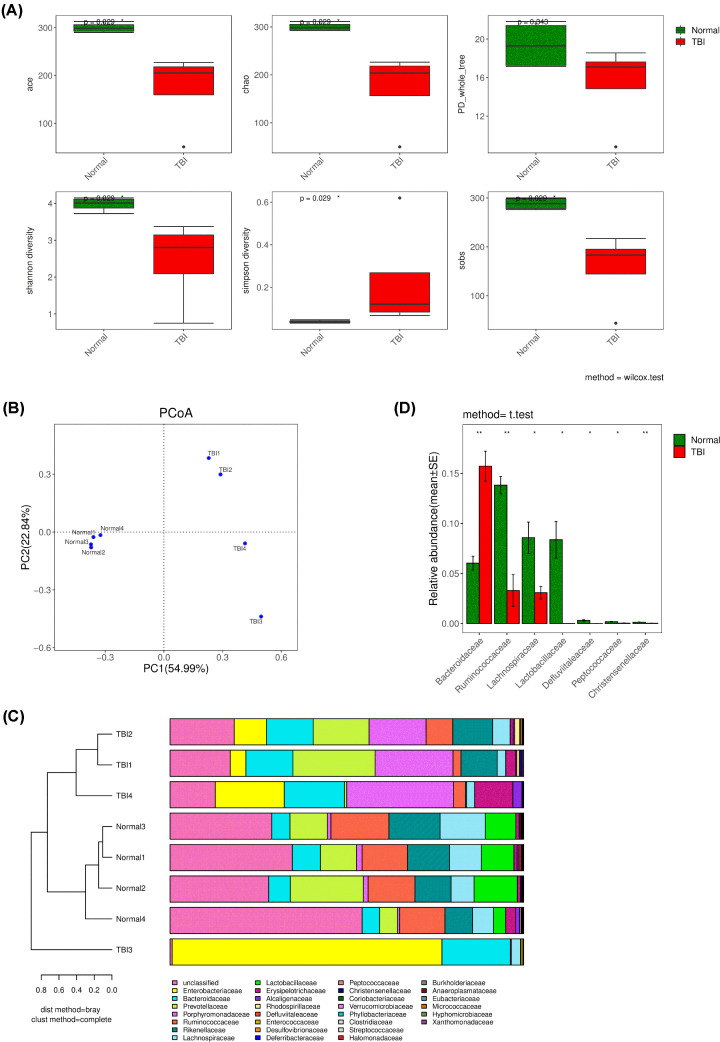
The gut bacterial diversity analysis and differential analysis of cecal contents from untreated or irradiated mice (**A**) ACE and Chao indices reflect species richness, Shannon and Simpson indices reflect species diversity (impacted by species richness and species evenness), and PD_whole_tree index is calculated from distance based on phylogenetic tree on operational taxonomic units (OTUs) level, and sobs: the observed species richness. Statistical significance was calculated using the Wilcoxon’s test and marked as the level of the *P* values. (**B**) The PCoA of microbial beta diversity indices at the OTU level in all groups. The percentages in the axis labels represent the percentages of variation explained by PCoA. (**C**) Combined analysis diagram of cluster tree and histogram of microbial community composition for microbial beta diversity in all groups on family level. (**D**) Differential analysis based on species information of bacteria in all groups on family level. Only abundant level > 0.5% was included and abundant level < 0.5% was identified as unclassified. Statistical significance was calculated using the *t* test and marked as the level of the *P-*values. *P<0.05, **P<0.01.

The bacterial diversity (Figure 5A), community distribution (Figure 5B), community composition (Figure 5C) and species differential analysis (Figure 5D) illustrated the TBI had obvious influence on intestinal bacteria. In general, the bacterial alpha and beta diversity decreased after TBI treatment compared with control group. The decreased diversity indicated the big change of intestinal micro-environment. Notably, the TBI impact on intestinal bacteria was stochastic since the normal groups gathered together but the TBI groups were discrete (Figure 5B). This stochastic influence caused by TBI may lead to complex consequence which will impact metabolic levels and further increase the difficulty of recovery. Uncovering the core species changes between normal and TBI groups may explain the mainly reasons that drive the changes of the intestinal micro-environment after TBI treatment. The differential analysis (Figure 5D) suggested that the *Bacteroidaceae, Ruminococcaceae, Lachnospiraceae, Lactobacillaceae, Defluviitaleaceae, Peptococcaceae* and *Christensenellaceae* were the major families that drove the bacterial diversity, distribution and even shaped the intestinal micro-environment after TBI treatment.

## Discussion

Acute radiation injury caused by high-dose radiation exposure severely limits the application of radiotherapy in cancer treatment and hematopoietic stem cell transplantation [3,4]. In our study, irradiation damaged intestinal mucosal barrier linked closely with decreased tight junction protein ZO-1 and pan-endothelial marker MECA32. However, the 4 Gy non-myeloablative irradiations increased MECA32 and ZO-1 at day 10 which was in line with IL-17/AhR expression. The damage of intestinal barrier after TBI was dose-dependent. Meanwhile, irradiations mainly decreased the protein expressions of P38 MAPK and increased the protein expressions of JNK MAPK signaling pathway. The irradiation reduced the total numbers of blood cells and MNCs in intestinal tract, and changed the percentages of various immune cells. Also, the irradiation changed SCFAs metabolism in cecal contents, especially under 8 Gy myeloablative irradiations. The irradiations induced low bacteria diversity and more community distribution compared with normal groups, which may explain the changes of SCFAs in the TBI-treated groups.

The intestinal epithelial cell barrier protects sterile micro-environments from physical, chemical and microbial challenge. Genotoxic stress by TBI affects intestinal stem cells, results in damage to the intestinal epithelial cell barrier, ultimately causes translocation of microbes to sterile compartments and subsequently changes immune activation [26]. The tight junction protein ZO-1 on the surface of intestinal epithelial cells is vital for gut integrity [16,27]. Epithelial tight junctions in the ileum and colon were disrupted by irradiation of 4 Gy with redistribution of ZO-1 from the intercellular junctions to the intracellular compartment [28]. Also, rapid angiogenesis could support nutrient for gut epithelium repairing after irradiation [29,30]. The localization of mouse pan-endothelial cell antigen MECA32 represents the continuity of vascular endothelium [31]. In our study, the 8 Gy myeloablative irradiations resulted in a significant reduction in intestinal ZO-1 expression. However, the 4 Gy non-myeloablative irradiations led to a slight decrease in intestinal ZO-1 protein at day 5 after TBI, followed by an increase at day 10. Likewise, MECA32 had higher level in the intestinal tract of 4 Gy mice at day 10, which suggested that the intestinal vascular epithelial cells were repaired after non-myeloablative radiation. Thus, accelerated angiogenesis might be a strategy to promote injured intestinal repairing.

The regeneration and apoptosis will happen caused by conditioning-induced gut damage. The stress-activated p38 MAPK has been implicated in gut injury, which acts as a pro-apoptotic cellular signaling during oxidative stress-induced intestinal epithelial cell injury [18,32]. However, cell migration via p38 MAPK/MAPK-activated protein kinase2/HSP27 pathway is critical for the regeneration in rapid repair of intestinal mucosal defects [33]. In our study, the p38-MAPK signal pathway was closely related to radio-induced intestinal injury. This can be explained by the decreased expression of ZO-1, MECA32 and phos-p38 after irradiation in the 4D10 group. Although mucosal injury repair was correlated with ERK1/2 activity and localization along the crypt–villus axis [17], the ERK1/2 MAPK signal pathway had no obvious correlation with 4/8 Gy-irradiatied mice. Furthermore, JNK is potentially activated by a variety of environmental stresses including γ irradiation. Our data also demonstrated 8 Gy TBI significantly up-regulate the level of JNK MAPK pathway than other groups.

SCFAs are the key metabolites generated by bacterial metabolism in the colon, which play key roles in intestinal repairing [16]. Butyrate is able to improve the homing T cells to gut mucosa by regulating molecules, thus maybe an important factor linking tight junctions between epithelial cells [16,34]. Oral gavage of valeric acid significantly ameliorated intestinal inflammation and dysfunction caused by 12 Gy total abdominal irradiation [27], because of valeric acid retains gut bacterial composition and restores epithelial integrity after irradiation. Our results showed that irradiations have negative effects on intestinal SCFAs depending on dosages. The bacterial diversity and key bacterial abundance decreased after irradiations. Meanwhile, the SCFAs related bacteria (*Bacteroidaceae* and *Ruminococcaceae*) were the key families to influence community distribution and micro-environment. Given these, the synbiotics therapy (with SCFAs) may be an effective way to treat acute intestinal tract radiation syndrome, improve intestinal micro-environment, and thus be beneficial for intestinal barrier repairing.

## Data Availability

Raw sequences of 16S rRNA gene amplicon sequencing were submitted to the National Center for Biotechnology Information as part of the BioProject PRJNA691429.

## Abbreviations

AhR: Aryl Hydrocarbon Receptor
DAPI: 4′,6-Diamidino-2-Phenylindole, Dihydrochloride
FDA: Food and Drug Administration
HRP: Horseradish peroxidase
IEL: intestinal intraepithelial lymphocyte
IL: interleukin
LN: lymph node
MAPK: mitogen-activated protein kinase
MNC: mononuclear cell
PLT: platelet
PMSF: Phenylmethanesulfonyl fluoride
P/S: penicillin/ streptomycin
RBC: red blood cell
RIPA: Radio Immunoprecipitation Assay
SAPK/JNK: stress-activated protein kinase/Jun-amino-terminal kinase
SCFA: short-chain fatty acid
TBI: total body irradiation
WBC: white blood cell
