# Correction: The effects of myeloablative or non-myeloablative total body irradiations on intestinal tract in mice

**DOI:** 10.1042/BSR-2020-2993_COR

**Published:** 2022-07-20

**Keywords:** intestinal tract, myeloablative irradiation, p38 MAPK, radiotherapy, short chain fatty acid, ZO-1

The authors of the original article “The effects of myeloablative or non-myeloablative total body irradiations on intestinal tract in mice” (*Biosci Rep* (2021) 41(3):BSR20202993. doi: 10.1042/BSR20202993). would like to correct Figure 2. Due to an error, they had placed an incorrect image in Figure 2A (ERK1/2 bands), and in Figure 2B (ERK1/2 gray levels). A revised version of Figure 2 is present in this Correction. The authors express their sincere apologies for any inconvenience that this error has caused to the readers. The authors declare that this Correction does not alter the conclusions of the study.

**Figure 2:**
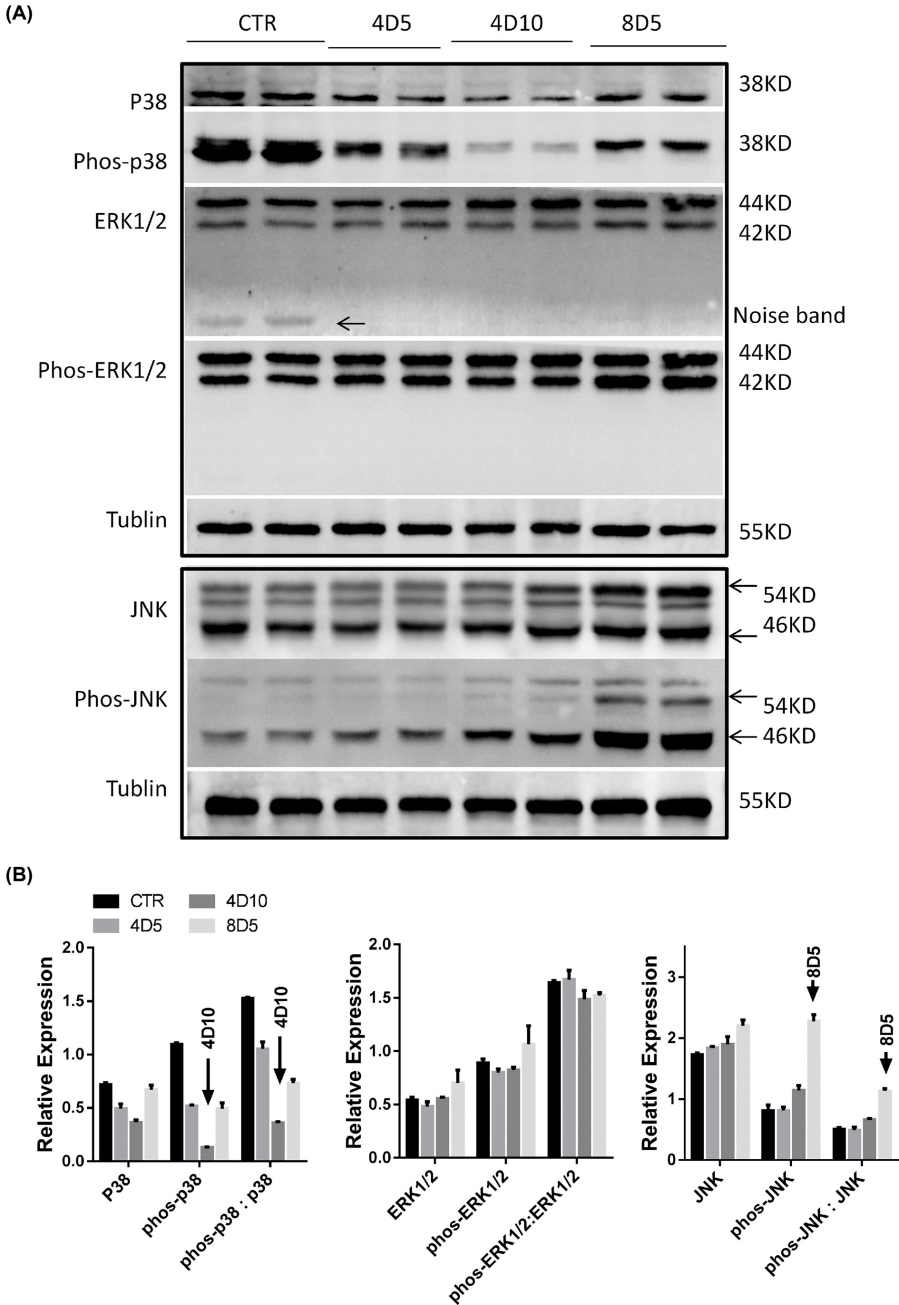
Irradiations affected the expression of intestinal MAPK signal pathway-related molecules (**A**) The different expressions of intestine tissue p38, phos-p38, ERK1/2, phos-ERK1/2, JNK and phos-JNK proteins in untreated or irradiated mice were analyzed by Western blotting. (**B**) Bar graph showed the gray levels of MAPK-related molecules as mentioned in (**A**).

